# Progressive balloon dilatation following hepaticojejunostomy improves outcome of bile duct stricture after iatrogenic biliary injury

**DOI:** 10.1186/1471-230X-13-70

**Published:** 2013-04-22

**Authors:** Zhu-lin Luo, Long Cheng, Jian-dong Ren, Li-jun Tang, Tao Wang, Fu-zhou Tian

**Affiliations:** 1 Department of General Surgery, General Hospital of Chengdu Military Command, Chengdu, Sichuan Province, People’s Republic of China; 2 Graduate School, Third Military Medical University, Chongqing, People’s Republic of China

**Keywords:** Iatrogenic biliary strictures, Cholecystectomy, Balloon dilation, Hepaticojejunostomy

## Abstract

**Background:**

Iatrogenic biliary stricture (IBS) is a disastrous complication of cholecystectomy. Although the endoscopic treatments are well accepted as initial attempts for IBS, surgical hepaticojejunostomy (HJ) is often necessary for a considerable proportion of patients. However, the anastomotic stricture after HJ also occurs.

**Methods:**

In the present study, a new procedure, progressive balloon dilation following HJ (HJPBD), was designed and utilized in the IBS treatment. We retrospectively compared HJPBD with the traditional HJ in term of the outcomes when used for IBS treatment.

**Results:**

Between January 1997 and December 2009, 112 patients with IBS attributed to cholecystectomy enrolled in our hospital were treated with surgical reconstruction with either HJ (n=58) or HJPBD (n=54). Of the 58 patients in HJ group, 48 patients (82.8%) had a successful outcome, while 52 out of 54 patients (96.3%) in HJPBD group achieved success. The successful surgical reconstruction rates were significantly different between these two groups, with a further improved outcome in patient undergone progressive balloon dilation following HJ. Additionally, 8 of the 10 failure cases in HJ group were successfully rescued by HJPBD procedure.

**Conclusions:**

Our findings suggest that the new procedure of HJPBD could be successfully applied to IBS patients, and significantly improve the outcome of IBS reconstruction.

## Background

Bile duct injury (BDI) occurs in a non-negligible proportion of patients who undergo cholecystectomy, especially laparoscopic cholecystectomy (LC) [1-3]. Delayed detection and inappropriate treatment of BDI post cholecystectomy are often complicated by biliary strictures. This type of bile duct stricture is treated non-surgically or by surgical hepaticojejunostomy (HJ). Endoscopic or radiologic interventions have often been initially attempted, but in vain in a considerable number of patients because of failure of guide-wire passage through the stricture site [4-6]. Even when the endoscopic procedure or radiologic interventions are successful, strictures recur in a considerable number of patients. So, at these circumstances, surgical hepaticojejunostomy become necessary.

The outcome of HJ for iatrogenic biliary stricture (IBS) is usually favorable, but a small proportion of patients suffer anastomotic stricture and recurrent bile duct stricture [7,8]. Thus, to reduce the occurrence rate of anastomotic stricture or recurrent bile duct stricture, several issues regarding HJ for IBS have been proposed and still waiting for solutions [9,10]. The questions that are often raised include: (1) when is the optimal time for operation (earlier or later?); and (2) which condition gives higher successful rate of HJ (disappearance of inflammatory edema or dilation of the proximal bile duct?). At present, HJ for IBS is often performed after the subsidence of inflammation and the dilation of proximal bile duct. The long-term success rates of HJ for IBS are reported ranging from 80%-90% [11,12]. Although only a small proportion of patients suffer failure, it remains unacceptable, because of the repeated cholangitis, biliary cirrhosis or even death just caused by what seems to be a simple operation, cholecystectomy.

In order to further improve the outcome of HJ for IBS, since 1997, we have designed and utilized a new procedure, progressive balloon dilatation following HJ (HJPBD), which was performed no mattering whether the bile duct is dilated or not [13]. In the current study, we retrospectively compare traditional HJ with HJPBD, in term of the technical complications and long-term outcome of patients with IBS. Our data showed that the new procedure HJPBD significantly improves outcome of IBS repair compared with traditional surgical procedures.

## Methods

### Patients

Between January 1997 and December 2009, 208 patients diagnosed with biliary strictures post-cholecystectomy were enrolled in a tertiary level referral hospital in China. In addition to ultrasonogram of the abdomen, endoscopic retrograde cholangiography, magnetic resonance cholangiography or percutaneous transhepatic cholangiography were performed for the assessment of strictures. Some patients had multiple cholangiograms. Ninety six patients with IBS who were managed conservatively or with endoscopic or radiological intervention as definitive treatment and achieved well outcome were excluded from this analysis. The remaining 112 patients who failed to achieve well outcome by endoscopic or radiological intervention or other conservative treatment were prepared to perform surgical reconstruction. Whether the traditional HJ or the new designed procedure HJPBD was performed was dependent on the maximal diameter of the upper segments of bile duct strictures. Accordingly, 58 patients with the maximal diameter of the upper segments more than 1.0 cm were performed with traditional HJ, and 54 patients with that less than 1.0 cm were performed with the new designed procedure HJPBD. All of these 112 patients underwent surgical reconstruction in the same Center of General Surgery of this hospital.

Ethical approval was given by the medical ethics committee of General Hospital of Chengdu Military Command area. All of the patients in the study were informed the possible complications brought by the surgical hepaticojejunostomy, and those patients underwent hepaticojejunostomy as well as balloon insert and fixation were informed the possible discomfort associated with the inserted balloons. The consent was obtained routinely before the surgical procedure and not for anything specifically related to this study.

### Surgical procedure

**Figure 1:**
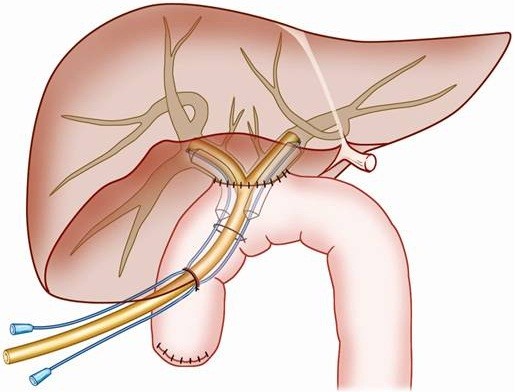
**Schematic diagram of ‘T’ tube and balloon placement.** The reconstructed hepatic bile duct was sutured with the jejunum by end-to-side anastomosis. The ‘T’ tube was inserted with each end extended into the left and right hepatic bile ducts respectively. The ‘T’ tube was fixed to the internal wall of the bile ducts. Balloons were inserted following ‘T’ tube placement and fixed to the ‘T’ tube. The balloons passed through the anastomotic stoma with one end in the enteric cavity and the other stretching into the left or right hepatic bile duct.

**Figure 2:**
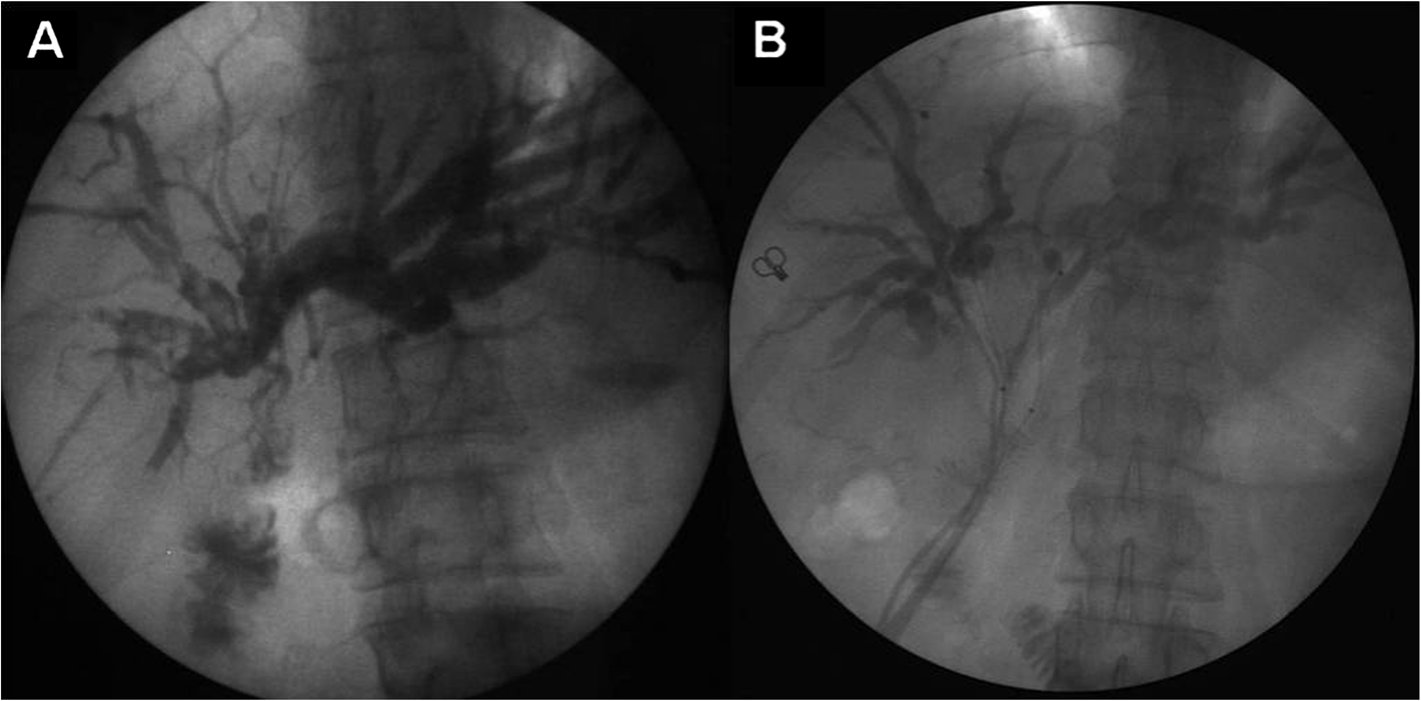
**Images of biliary system before and after HJPBD. A**: Before HJPBD, the X-ray photography with cholangiography showed that the lesions located at the common hepatic duct stump with the ceiling of the confluence was destroyed; **B**: Two months after HJPBD, the X-ray photography with cholangiography showed that the two balloons were still at their positions, both passing through the anastomotic stoma with one end in the enteric cavity and the other stretching into the left or right hepatic bile duct. HJPBD: progressive balloon dilation following hepaticojejunostomy.

Roux-en-Y HJ was the standard surgical repair. In patients treated with abdominal drainages, definitive operations were performed after 1 month’ drainages. During the operation, scars and granulation tissues of the injured bile duct were dissected, the distal end of the injured bile duct was closed, and the intact proximal end of the bile duct was opened with "Y"-shape incision which extended to the left and right hepatic bile ducts. Through the "Y" incision, three duct branches could be seen (two of the right hepatice bile ducts and one of the left hepatic bile duct). The reconstructed hepatic bile duct was then sutured with the jejunum by end-to-side anastomosis. A “T” stent was placed into the left and right hepatic bile ducts and sewed on internal wall of the bile ducts to ensure its position for 6 months. In HJPBD group, two trans-anastomotic balloons were inserted and fixed with the "T" stent, Figures 1 and 2.

### Progressive balloon dilation

The balloon (Cook Inc., Bloomington, IN, USA) placed at the anastomosishas a length of 30-mm in column shape with a diameter of 1.8 mm and 10 mm before and after insufflation, respectively. One week after operation, the balloon was insufflated in a slow pace to avoid laceration to the bile duct. Dilation was done 4 times each day and maintained for 2 to 4 hours each time. The initial volume of insufflations varied due to different severity of biliary stricture. The volume and pressure required for insufflation were mainly based on the subjective tolerance of the pain caused by dilatation, and gradually reached to the maximal volume of about 10 ml within 2 months post-operation. During the third month post-operation, the balloon insufflation was kept with the maximal volume for 4 hours each time and 4 times per day. In this way, the bile duct was kept in a dilated state in 2/3 time daily, and in the rest of time the balloon was decompressed to let the bile draining. The patients were trained to be familiar with the performance of dilatation during hospitalization, and the dilatation should be performed by patients themselves out of hospitalization. At the end of the third month after surgical reconstruction, the "T" stent and decompressed balloon were removed after checking the anastomosis junction and portal bile duct using cholangiography.

### Follow-up data and evaluation criteria

Follow-up information was collected by outpatient visits, mailed questionnaires, and telephonic interviews. Follow-up evaluation was done by clinical history and examination, liver function tests (LFT), and US. Patients who were eligible for a minimum follow-up of 2 years at the time of analysis (Dec 2011) were included in the study. Outcome of surgical repair was stratified into four grades as previously described by McDonald [14]: Grades A (asymptomatic, normal LFT), B (asymptomatic, mild LFT derangement), C (pain, cholangitis defined as fever with jaundice, and abnormal LFT), and D (surgical revision or dilatation required). Patients with grade A and B were classified as treatment successes, while patients with grade C and D were classified as failures.

### Statistical analysis

Statistical analyses were performed using SPSS version 13.0 for Windows (Statistical Package for the Social Sciences; SPSS, Munich, Germany). Continuous variables in the 2 groups were analyzed with a Student *t* test or Mann–Whitney *U* test according to the continuous data with normal or non-normal distributions. Differences in categorical variables were analyzed by *χ*^2^ and Fisher’s exact tests. A 2-tailed *P* value < 0.05 was considered statistically significant.

## Results

### Patient characteristics

One hundred and twelve patients with IBS were included in the present study. There was no difference in baseline characteristics between HJ and HJPBD groups, Table 1. Of them 51 patients were males and 61 were females aged from 13 to 74 years old. In the 112 patients, a half of patients (56 out of 112) have not suffered episode of cholangitis indicating by lack of chills, fever and jaundice. Of the 112 IBS patients, 29 had undergone open cholecystectomy (OC) and 83 had undergone laparoscopic cholecystectomy (LC).

**Table 1:** Baseline characteristics

	**HJ**	**HJPBD**
N	58	54
Sex, F/M	33/25	28/26
Age, years (mean± SD)	42.6±9.4	39.8±8.5
Cholecystectomy, OC/LC	16/42	13/41
Early detection of bile duct injury	36	30
Episode of cholangitis	29	27
Median time from cholecystectomy to biliary stricture, months	9.5	11.0
Type of biliary stricture		
Type I	14	14
Type II	13	9
Type IIIa	10	11
Type IIIa	18	19
Type IV	3	1
Undergone ERCP or PTC before surgery	47	44

Notes: There was no difference in baseline characteristics between HJ and HJPBD groups.

Bile duct injuries were observed in 66 patients within 7 days post-cholecystectomy, and had been initially treated with T tube drainage (n =23), bile duct end-to-end anastomosis (n=31) or local tissue repairs (n =12). The strictures were detected at 3 to 43 months after cholecystectomy. Ninety one patients with stricture were undergone at least one attempt of ERCP or PTC. Bile duct strictures were classified into five types according to their location in relationship to the hepatic duct bifurcation as previously described by Bismuth [15].

### Outcomes of patients

Median follow-up was 52 (24– 156) months. There was not any significant difference between HJ and HJPBD groups regarding to operation duration time, bleeding volume, anesthesia, etc. Outcome of surgical repair was stratified into four grades as previously described by McDonald [14], and the patients with grade A and B were classified as treatment successes, while patients with grade C and D were classified as failures. Of the 58 patients in HJ group, 48 patients (82.8%) had successful outcome, while 52 out of 54 patients (96.3%) in HJPBD group achieved success. Moreover,in long-term follow-up, patients achieving grades A (asymptomatic, normal LFT) account more in HJPBD group than that in HJ group, Table 2. Statistic analysis showed that the successful surgical reconstruction rates were significantly different between these two groups (p<0.05), with a further improved outcome in patient undergone progressive balloon dilation following HJ.

**Table 2:** General outcome of patients

		**HJ**	**HJPBD**
Successes	Grade A	36	45
	Grade B	12	7
Failures	Grade C	4	1
	Grade D	6	1
Total	58	54	

Notes: Outcome of surgical repair was stratified into four grades as previously described by McDonald [14]: Grades A (asymptomatic, normal LFT), B (asymptomatic, mild LFT derangement), C (pain, cholangitis defined as fever with jaundice, and abnormal LFT), and D (surgical revision or dilatation required). Patients with grade A and B were classified as treatment successes, while patients with grade C and D were classified as failures.

Additionally, of the 10 failure cases in HJ group, 1 patient suffered death (due to repeated episodes of cholangitis and subsequent liver cirrhosis), and the other 9 patients received HJPBD procedure with 8 patients successfully rescued. Overall the procedure of HJPBD failed in 3 patients, of whom one received PTC, one underwent second HJPBD and another underwent transplantation.

The occurrence of biliary complications after surgical reconstruction was also analyzed. While the incidences of cholangitis, bile leakage and biliary bleeding were comparable between the two groups, the restenosis occurred more frequently in HJ group than in HJPBD group (12.07% versus 1.85%), Table 3. The results suggested that the performance of the new procedure could reduce the incidence of bile duct restenosis without increasing the other biliary complications.

**Table 3:** Biliary complications post surgical reconstruction

**Complications**	**HJ (n=58)**	**HJPBD (n=54)**	**P value**
**Cholangitis**	5	2	>0.05
**Bile leak**	3	4	>0.05
**Biliary bleeding**	1	1	>0.05
**Restenosis**	7	1	<0.01

## Discussion

Since the widespread of LC, iatrogenic biliary stricture (IBS) due to cholecystectomy appears more frequently, and its treatment becomes a challenging topic for hepatobiliary surgeons. Although endoscopic treatments play an important role in the IBS therapy, a considerable portion of patients still need surgical hepaticojejunostomy (HJ) [16]. At present, HJ for IBS is often performed after the subsidence of inflammation and the dilation of proximal bile duct [15,17]. Subsidence of inflammation is necessary for operation, and one month is often sufficient for the inflammation recovery through appropriate treatment. However, it remains to be addressed how to gain satisfactory dilation and whether it is feasible to perform HJ without dilation of proximal bile duct.

It is generally believed that the extra-hepatic bile duct with smaller diameter is more prone to be injured during cholecystectomy [18,19]. For these patients, the success rate of HJ is relatively lower if the proximal bile duct segments of the stenosis are not dilated, circumstances that are present in more than half of patients. So it is necessary to take proper steps to gain satisfactory dilation for these patients. However, it is difficult to obtain a satisfactory dilated bile duct (>10mm) in clinical practice. As forceful dilation of bile duct during operation is not practical since it is easy to lacerate the bile duct, 3 to 6 months’ passive dilatation before operation is recommended to make it easy for anastomosis and to reduce the recurrence rate of strictures [20]. However, the long time waiting for the dilation is sometimes unacceptable, and during this period, more treatments will be proposed. Even if patients could endure to the end of the procedure for the dilation of the bile duct, there are still a considerable number of patients that could not be beneficiated with satisfactory dilation of bile ducts.

To avoid the passive waiting for the dilation of bile duct before HJ, we designed a positive dilation procedure after HJ, based on the principle of subcutaneous balloon dilation to obtain skin flap in the facial orthopedic operation. In the current study, we performed HJ after the subsidence of inflammation, no mattering whether the bile ducts were dilated or not dilated, and simultaneously inserted a flexible balloon, which could progressively dilate the anastomotic site. With a follow-up of 2 to 13 years, we observed a better long-term outcome for IBS treatment with this new procedure comparing with traditional HJ. We found that the anastomosis site can be dilated enough by progressively dilating with the balloon for three months after surgery. Additionally, we found the anastomosis site would not shrunk back after dilated, probably because of the balloon prop during the period of wound healing and tissue remodeling at the site of anastomosis. However, this exciting outcome need to be further confirmed by following randomized-controlled trial which had been initiated by us. Meanwhile, the role and mechanisms of progressive balloon dilation in bile duct tissue remodeling should be further investigated in animal models.

Complications of progressive balloon dilation include balloon loosing, broken and air leaking, which all influence the outcome of IBS treatment. Therefore, a careful caution should be taken during the procedure. Since subjective turgid feeling is the best indication to control the volume for balloon insufflation, patients should be informed how to do balloon insufflations. Patients could insufflate balloon by themselves and control insufflation volume and pressure based on their own feelings. In addition, patients should know how to take care of the balloon to avoid slippage or damage to it.

## Conclusions

In conclusion, our findings suggest that the new procedure, i.e. earlier hepaticojejunostomy followed by progressive balloon dilation, could be successfully applied to IBS patients and significantly improved the outcome of IBS reconstruction. Based on our obseration, we tentatively proposed that the optimal operation timing for successful IBS reconstruction may be as early as the subsidence of inflammation, instead of waiting for the dilatation of the proximal segment of stricture.

## Consent

Written informed consent was obtained from the patients for publication of this report and any accompanying images.

## Competing interests

The authors declare that they have no competing interests.

## Pre-publication history

The pre-publication history for this paper can be accessed here:

http://www.biomedcentral.com/1471-230X/13/70/prepub

## References

[B1] Savader SJ Lillemoe KD Prescott CA Winick AB Venbrux AC Lund GB Mitchell SE Cameron JL Osterman FA Jr Laparoscopic cholecystectomy related bile duct injuries: a health and financial disaster Ann Surg 1997 225 3 268 273 10.1097/00000658-199703000-00005 9060582 PMC1190676

[B2] Pesce A Portale TR Minutolo V Scilletta R Li Destri G Puleo S Bile duct injury during laparoscopic cholecystectomy without intraoperative cholangiography: a retrospective study on 1,100 selected patients Dig Surg 2012 29 4 310 314 10.1159/000341660 22986956

[B3] Richardson MC Bell G Fullarton GM Incidence and nature of bile duct injuries following laparoscopic cholecystectomy: an audit of 5913 cases Br J Surg 1996 83 10 1356 1360 10.1002/bjs.1800831009 8944450

[B4] Jabłońska B Lampe P Iatrogenic bile duct injuries: etiology, diagnosis and management World J Gastroenterol 2009 15 33 4097 4104 10.3748/wjg.15.4097 19725140 PMC2738802

[B5] Kassab C Prat F Liguory C Meduri B Ducot B Fritsch J Choury AD Pelletier G Endoscopic management of post-laparoscopic cholecystectomy biliary strictures: Long-term outcome in a multicenter study Gastroenterol Clin Biol 2006 30 1 124 129 10.1016/S0399-8320(06)73127-X 16514393

[B6] Misra S Melton GB Geschwind JF Venbrux AC Cameron JL Lillemoe KD Percutaneous management of bile duct strictures and injuries associated with laparoscopic cholecystectomy: a decade of experience J Am Coll Surg 2004 198 2 218 226 10.1016/j.jamcollsurg.2003.09.020 14759778

[B7] Lillemoe KD Melton GB Cameron JL Pitt HA Campbell KA Talamini MA Sauter PA Coleman J Yeo CJ Post operative bile duct strictures: management and outcome in the 1990s Ann Surg 2000 232 3 430 441 10.1097/00000658-200009000-00015 10973393 PMC1421156

[B8] Sikora SS Pottakkat B Srikanth G Kumar A Saxena R Kapoor VK Postcholecystectomy benign biliary strictures – long-term results Digest Surg 2006 23 5–6 304 312 10.1159/000097894 17164542

[B9] Laasch HU Martin DF Management of benign biliary strictures Cardiovasc Intervent Radiol 2002 25 6 457 466 10.1007/s00270-002-1888-y 12391514

[B10] Pottakkat B Vijayahari R Prakash A Singh RK Behari A Kapoor VK Saxena R Factors predicting failure following high bilio-enteric anastomosis for after Iatrogenic Biliary Injury induced by cholecystectomy benign biliary strictures J Gastrointest Surg 2010 14 9 1389 1394 10.1007/s11605-010-1241-8 20589447

[B11] Costamagna G Shah SK Tringali A Current management of postoperative complications and benign biliary strictures Gastrointest Endosc Clin N Am 2003 13 4 635 648 10.1016/S1052-5157(03)00103-X 14986791

[B12] Chaudhary A Negi SS Puri SK Narang P Comparison of magnetic resonance cholangiography and percutaneous transhepatic cholangiography in the evaluation of bile duct strictures after cholecystectomy Br J Surg 2002 89 4 433 436 10.1046/j.0007-1323.2002.02066.x 11952583

[B13] Tian FZ Tan LJ Luo H Li KZ Wang Y Li DX Progressive balloon dilatation following hepaticojejunostomy in the treatment of traumatic biliary strictures Chin J Dig Surg 2009 8 1 18 20

[B14] Zepeda-Gómez S Baron TH Benign biliary strictures: current endoscopic management Nat Rev Gastroenterol Hepatol 2011 8 10 573 581 10.1038/nrgastro.2011.154 21894200

[B15] Bismuth H Majno PE Biliary strictures: classification based on the principles of surgical treatment World J Surg 2001 25 10 1241 1244 10.1007/s00268-001-0102-8 11596882

[B16] Rodriguez-Montes JA Rojo E Martin LG Complications following repair of extrahepatic bile duct injuries after blunt abdominal trauma World J Surg 2001 25 10 1313 1316 10.1007/s00268-001-0116-2 11596896

[B17] Vinay K Kapoor: bile duct injury repair: when? what? who? J Hepatobiliary Pancreat Surg 2007 14 5 476 479 10.1007/s00534-007-1220-y 17909716

[B18] Club TSS A prospective analysis of 1518 laparoscopic cholecystectomies N Engl J Med 1991 324 16 1073 1078 1826143 10.1056/NEJM199104183241601

[B19] Karvonen J Salminen P Grönroos JM Bile duct injuries during open and laparoscopic cholecystectomy in the laparoscopic era: alarming trends Surg Endosc 2011 25 9 2906 2910 10.1007/s00464-011-1641-1 21432006

[B20] Nealon WH Urrutia F Long-term follow-up after bilioenteric anastomosis for benign bile duct stricture Ann Surg 1996 223 6 639 648 10.1097/00000658-199606000-00002 8645037 PMC1235203

